# Atraumatic Versus Silver‐Modified Atraumatic Restorative Treatment in Primary Molars: A Randomized Clinical Trial on Minimally Invasive Caries Management and Oral Health‐Related Quality of Life

**DOI:** 10.1002/cre2.70299

**Published:** 2026-02-03

**Authors:** Sana K. Solh, Ahmed A. Holiel, Ahmad S. Tarabaih

**Affiliations:** ^1^ Division of Pediatric Dentistry, Faculty of Dentistry Beirut Arab University Beirut Lebanon; ^2^ Conservative Dentistry Department, Faculty of Dentistry Alexandria University Alexandria Egypt; ^3^ Department of Restorative Sciences, Faculty of Dentistry Beirut Arab University Beirut Lebanon

**Keywords:** atraumatic restorative treatment, early childhood caries, oral health‐related quality of Life, primary molars, silver diamine fluoride, silver‐modified atraumatic restorative treatment

## Abstract

**Objectives:**

To compare the 6‐month clinical success of atraumatic restorative treatment (ART) and silver‐modified atraumatic restorative treatment (SMART) in primary molars of children with early childhood caries (ECC), and to assess the impact of these treatments on oral health‐related quality of life (OHRQoL).

**Materials and Methods:**

A randomized controlled split‐mouth trial included 32 children (aged 3–7 years) with 68 primary molars exhibiting active dentin carious lesions (ICDAS II scores 4 or 5). Each child received one ART restoration using high‐viscosity glass ionomer cement and one SMART restoration with silver diamine fluoride, followed by HVGIC in the same session. Clinical success was assessed after 6 months, using modified ART criteria. Parents completed the Arabic version of the Early Childhood Oral Health Impact Scale (A‐ECOHIS) at baseline and after 6 months to assess changes in OHRQoL. Data analysis included chi‐square, Fisher's exact test, Student's *t*‐test with effect sizes reported, and a significance level set at 95%.

**Results:**

Of the 68 restorations (34 ART, 34 SMART), 6‐month success rates were 67.6% for ART and 70.5% for SMART, with no statistically significant difference (*p* = 0.66). SMART showed slightly better caries arrest. Class I restorations had higher success rates than Class II for both techniques. Failures were mainly due to wear and marginal integrity loss. Mean ECOHIS scores improved from 16.9 at baseline to 10.13 at 6 months, though the change was not statistically significant (*p* = 0.125).

**Conclusion:**

SMART and ART techniques showed similar short‐term clinical outcomes, with SMART showing a minor, nonsignificant advantage in caries management. Failures in ART were more often linked to active caries and pulp involvement, suggesting that SMART may enhance caries arrest. Placement of both restorations did not significantly affect OHRQoL.

**Clinical Significance:**

Incorporating SDF may improve caries arrest and the effectiveness of GIC restorations in primary molars.

**Trial Registration:**

ClinicalTrials.gov identifier: NCT07023939.

## 1 Introduction

Early childhood caries (ECC) is one of the most widespread childhood diseases, affecting 60%–90% of children worldwide (World Health Organization 2017), particularly those from underprivileged backgrounds with limited access to dental care (Heinrich‐Weltzien et al. 2013). Untreated ECC can have profound and lasting effects on oral function, nutrition, and quality of life (Northridge et al. 2020).

Minimal intervention dentistry (MID) has significantly transformed the management of dental caries, particularly in young children (Wakhloo et al. 2021). It is recognized by the American Academy of Pediatric Dentistry (AAPD) as an essential component of comprehensive patient care, as it helps preserve tooth structure, delay the restorative cycle, and maintain functional dentition (American Academy of Pediatric Dentistry 2023).

The atraumatic restorative treatment (ART) approach is a well‐established minimally invasive technique commonly used to manage cavitated carious lesions in both clinical and community settings (Dipalma et al. 2025). When compared to the traditional drilling method, ART is more comfortable and better tolerated by young children, as it causes less pain and dental anxiety (Frencken 2017). However, concerns remain regarding residual cariogenic bacteria beneath the restorations (Hafshejani et al. 2017) and the relatively lower survival rate of multi‐surface restorations in primary teeth (Giacaman et al. 2018).

Silver diamine fluoride (SDF) is another well‐accepted minimally invasive approach for managing cavitated carious lesions (Hansen et al. 2019). Recently, the AAPD strongly recommended the use of 38% SDF as part of a comprehensive management plan to arrest active lesions on primary teeth, particularly for uncooperative, young, or special health care needs children (American Academy of Pediatric Dentistry 2024).

However, one major drawback of SDF is the black staining of treated lesions, which may cause esthetic concerns (Crystal and Niederman 2019). Furthermore, while SDF can arrest caries progression, the cavity remains unfilled, potentially compromising plaque control and chewing ability (Duangthip et al. 2018).

Silver‐modified atraumatic restorative treatment (SMART) is a novel approach that combines the antibacterial properties of SDF with the sealing ability of glass ionomer cement (GIC) (Amend et al. 2022). This method not only arrests caries but also enhances enamel remineralization, while preserving pulp vitality by sealing the cavity and restricting bacterial growth through nutrient deprivation (Alvear et al. 2016).

Recently, patient‐reported outcomes have gained importance alongside clinical results (Kalenderian et al. 2024). Oral health‐related quality of life (OHRQoL) is a multidimensional concept reflecting the impact of oral health on daily life (BaniHani et al. 2018). Prior research suggests that ECC negatively affects OHRQoL in young children (Zaror et al. 2022). The impact of ECC on OHRQoL is evaluated using standardized scales. For children of preschool age and younger, the ECOHIS (Early Childhood Oral Health Impact Scale) is the most commonly used (Farsi et al. 2018).

The aim of the current study was to compare the clinical outcomes of ART and SMART restorations in terms of wear, marginal integrity, retention, caries arresting, and pulpal pathology, while also evaluating changes in OHRQoL before and after treatment. Two null hypotheses were tested: (1) There is no difference in the clinical performance of SMART and ART restorations in primary molars. (2) There is no significant difference in the OHRQoL scores before and after treatment by these restorations.

## 2 Materials and Methods

### 2.1 Subjects, Study Design, and Setting

This was a randomized, split‐mouth design clinical trial conducted at the Pediatric Dentistry outpatient clinic of Beirut Arab University between February 2022 and December 2023. The Consolidated Standards of Reporting Trials (CONSORT) guidelines for clinical trials were followed (Schulz et al.^2010^). The study adhered to the ethical guidelines outlined in the Declaration of Helsinki and obtained ethical approval from the university's Institutional Review Board (IRB code: 2022‐H‐011‐D‐M‐0489). Written informed consent was obtained from parents or guardians after explaining the study's objectives, benefits, and risks in simple Arabic. The trial was also registered at ClinicalTrials.gov (Identifier: NCT07023939).

### 2.2 Inclusion and Exclusion Criteria

The study sample included 34 healthy children, aged 3–7 years, exhibiting either negative (Score 2) or positive (Score 3) behavior according to Frankl's behavior rating scale (Frankel and Shiere 1962).

Children of both genders were eligible for the study if they had at least two bilateral, asymptomatic decayed primary molars with active occlusal or occluso‐proximal lesions, scored as 4 or 5 according to the International Caries Detection and Assessment System (Ismail et al. 2007).

Children were excluded from the study if they had systemic diseases or conditions classified as ASA Type I, or if their behavior was rated as definitely positive (Score 1) or definitely negative (Score 4) according to Frankel's behavior rating scale. Molars were also excluded if they had existing restorations, pulpal pathology (abscess, fistula, and mobility), or extensive structural loss (ICDAS 6 or deep cavities).

### 2.3 Sample Size Calculation and Randomization

The sample size was specified by using the G* power test, assuming a statistical level of significance of 95% and power of 80% with normal distribution, and effect size calculated from the means and standard deviation of two groups obtained from a previous similar study (Jiang et al. 2020). The original sample size was 64 molars and was increased to 80 to avoid any drop‐out.

Using a simple randomization method with an allocation ratio of 1:1, eligible molars were randomly and equally assigned to either the SMART or ART group using a randomization software (Random.org).

### 2.4 Calibration

One calibrated examiner was responsible for all the dental examinations. The intra‐examiner agreement was assessed by re‐examining 10% of the sample. The Kappa statistic for intra‐examiner reliability was 0.87, which indicates consistency in the application of scoring criteria over the study period.

### 2.5 Interventions

Before starting treatment, all participants were given oral hygiene instructions and dietary advice. All restorations were performed by the same operator.

### 2.6 ART Group

Partial isolation was achieved using cotton rolls and saliva ejectors. The cavity and adjacent tooth surfaces were cleaned with a low‐speed brush. The treatment was conducted as described by Holmgren et al. (2013) as follows: Selective caries removal was done using hand instruments—carious dentin was excavated from the axial walls with a spoon excavator, and unsupported enamel was carefully removed using a hatchet. On the pulpal floor, only soft, completely demineralized dentin was removed until firm dentin was reached.

### 2.7 Smart Group

Similar isolation and cleaning protocols were followed. To improve chemical bonding with the GIC, soft caries were selectively removed from cavity margins as described by Alvear et al. (2016). The cavity was dried with compressed air while maintaining isolation. SDF was applied according to the AAPD's Chairside Guide on Silver Diamine Fluoride in the Management of Dental Caries Lesions (AAPD 2017) as follows: (1) the surrounding gingival tissues and lips were protected with petroleum jelly to avoid staining and irritation (2) one drop of SDF (Advantage Arrest, Elevate Oral Care, USA) was placed into a disposable dish, (3) SDF was applied with a bended micro‐sponge directly to the affected tooth surfaces for minimum of 60 s, (4) excess was blotted with a cotton pellet, and (5) the area was air‐dried while maintaining isolation for up to 3 min.

### 2.8 For Both Groups

The cavity and surrounding surfaces were conditioned by diluting the liquid component of the GIC, as described by Holmgren et al. (2013). This involved moistening a cotton pellet with water, blotting it dry, then dipping it in the GIC liquid. The conditioner was applied for 15 s and rinsed off after 10 s. GIC was mixed per manufacturer instructions and applied in small increments using the rounded end of a large excavator. The restoration was contoured with a carver, excess material was removed, and petroleum jelly was applied to cover the surface. Patients were advised not to eat for at least 1‐h post‐treatment.

### 2.9 Blinding

Since the current study was an interventional study, the operator could not be blinded to the treatment method used. During follow‐ups, complete blinding was not feasible in some cases, particularly where GIC restorations were lost due to noticeable staining of the cavity with SDF. However, blinding of the data analyst was done, where all study data were labeled with non‐identifying Alphabet letters to reduce the risk of bias.

### 2.10 Outcome Assessment

Follow‐up assessments were done by the operator at 6 months. The status of the restorations and restored teeth was assessed clinically and recorded using modified ART restoration criteria commonly adopted in previous clinical study of ART restorations (Duangthip et al. 2018) (Table 1). Clinical examination was carried out using plane mouth mirrors and CPI probes. Scores 0 and 1 indicate the success of the restoration, while scores ranging from 2 to 9 are considered as failures.

**Table 1:** Scores of modified ART restoration criteria.

Score 0	Restoration present, no caries, no marginal defects, or wear
Score 1	Restoration present, no active caries, slight defects or wear not greater than 0.5 mm
Score 2	Restoration present, marginal defects greater than 0.5 mm
Score 3	Restoration present, wear greater than 0.5 mm
Score 4	Restoration present, active caries found associated with the filling
Score 5	Restoration missing, no active caries, surface hard to gentle probing
Score 6	Restoration missing, active caries found, surface soft to probing, allowing penetration of the tip of a blunt probe
Score 7	Tooth with signs of pulpal pathology
Score 8	Tooth missing, extracted due to caries
Score 9	Tooth naturally exfoliate
Outcome	Success 0–1
	Failure 2–9

Wear, active, and arrested caries were determined by the visual and tactile examination method used by Young et al. (2015). Marginal Integrity was evaluated by visual Inspection and explorer examination as described by Frencken and Holmgren (1999).

Active and arrested caries were evaluated through visual inspection and tactile examination (ADA 2015).

### 2.11 Data Collection

At baseline, parents completed a two‐part questionnaire: Part 1 gathered general demographic information, while Part 2 included the validated Arabic version of the Early Childhood Oral Health Impact Scale (A‐ECOHIS) validated by Farsi et al. (2017). At the second follow‐up visit, parents completed the same questionnaire again.

### 2.12 Statistical Analysis

Data were analyzed using SPSS (version 28, IBM SPSS Statistics, USA). The Shapiro‐Wilk test indicated that most variables were normally distributed. A significance level of *p* ≤ 0.05 was applied throughout. Changes in ECOHIS scores before and after intervention were assessed using the Paired Samples t‐test. Fisher's exact test and Chi‐Square test were used to compare outcomes between ART and SMART at single time points, while the relationship between treatment success and cavity type was analyzed using the chi‐square test of Independence.

## 3 Results

**Figure 1:**
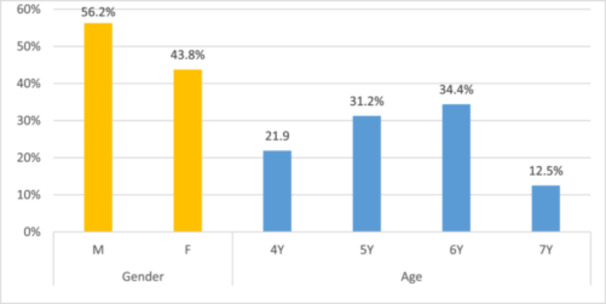
Participants distribution according to gender and age.

A total of 34 children were enrolled and received the interventions. During the follow‐up period, two participants were lost due to lack of compliance. Consequently, 32 children completed the study, including 18 males (56.2%) and 14 females (43.8%), with a mean age of 5.35 (±0.95), ranging from 4 to 7 years old (Figure 1).

**Figure 2:**
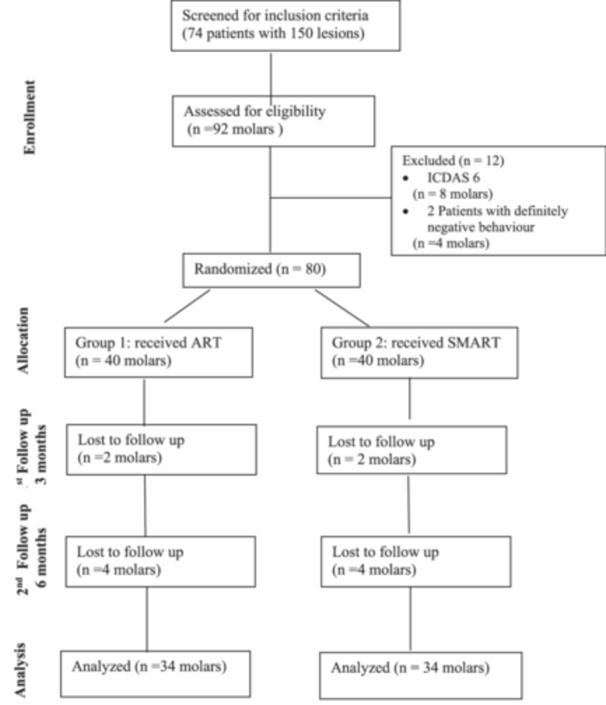
CONSORT flowchart.

As a result, 68 molars (85%) from 32 children were available for the final clinical analysis. No replacement or additional recruitment was undertaken. The flow of molars through the study is illustrated in Figure 2.

### 3.1 Overall Success and Failure Rates at 6 Months

At 6 months, the success rate for SMART is 70.5%, which is higher than ART at 67.6%. The *p*‐value of 0.663 suggests no statistically significant difference in outcomes between the two treatments (Table 2).

**Table 2:** Overall success and failure rates in ART and SMART restorations at 6 months.

		Outcome at 6 months				*p*
		Success		Failure		
		*n*	%	*n*	%	
Treatment	ART	23	67.60	11	32.40	0.663
	SMART	24	70.50	10	29.50	

*Note:* Chi‐square test.

### 3.2 Clinical Performance of ART and SMART at 6 Months

**Figure 3:**
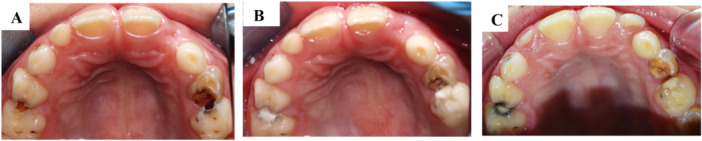
Treatment and follow‐up photos for Class II cavities showing success and failure patterns. (A) Pre‐operative photo for Class II cavities in teeth #55 and #65. (B) Post‐operative photo. (C) 6‐month follow‐up: Tooth #55 showing wear and loss of marginal integrity. Tooth #65 showing success as wear was less than 0.5 mm.

Table 3 shows the clinical performance of ART and SMART at 6 months; the ART and SMART treatments performed almost similarly, with no statistically significant differences in terms of marginal defects or wear (*p* values = 1.000) (Figures 2 and 3).

**Table 3:** The clinical performance of ART and SMART at 6 months.

		6 months follow‐up				*p*
		ART		SMART		
		*n*	%	*n*	%	
Score 0	Restoration present, no caries, no marginal defects, or wear	4	11.8	6	17.6	0.734 a
Score 1	Restoration present, no active caries, slight defects or wear not greater than 0.5 mm	19	55.9	18	52.9	1.000 a
Score 2	Restoration present, marginal defects greater than 0.5 mm	2	5.9	2	5.9	1.000 a
Score 3	Restoration present, wear greater than 0.5 mm	4	11.8	2	5.9	0.673 a
Score 4	Restoration present, active caries found associated with the filling	2	5.9	0	0.0	0.493 a
Score 5	Restoration missing, no active caries, surface hard to gentle probing	1	2.9	6	17.6	0.105 a
Score 6	Restoration missing, active caries found, surface soft to probing, allowing penetration of the tip of a blunt probe	1	2.9	0	0.0	1.000 a
Score 7	Tooth with signs of pulpal pathology	1	2.9	0	0.0	1.000 a
Score 8	Tooth missing, extracted due to caries	0	0.0	0	0.0	—
Score 9	Tooth naturally exfoliate	0	0.0	0	0.0	—
Outcome	Failure	11	32.4	10	29.5	1.000 b
	Success	23	67.6	24	70.5	

^a^ Fisher's exact test.

^b^ Chi‐square test.

**Figure 4:**
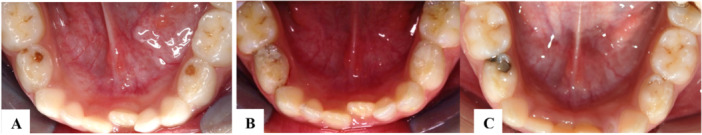
Treatment and follow‐up photos for Class I cavities showing failure patterns. (A) Pre‐operative photo for Class I cavities on tooth #74 and #84. (B) Post‐operative photo. (C) 6‐month follow‐up: Tooth #74 showing wear more than 0.5 mm, and tooth #84 showing wear and marginal defects more than 0.5 mm.

However, a notable difference was observed concerning caries management. In the ART group, active caries was associated with 5.9% of restorations where the filling was present, and in 2.9% of cases where the restoration was missing, with pulp pathology also emerging in 2.9% of cases. In contrast, no active caries was detected in SMART restorations, even when the restoration was missing, which occurred in 17.9% of cases (Table 3, Figures 3 and 4).

### 3.3 Success and Failure Rates in ART and SMART Restorations Among Class I and Class II Cavities

The success rate for both restorations was associated with the class of restorations. Class I restorations had the highest success rate with 80% for SMART and 76.9% for ART, followed by class II restorations with 66.6% for SMART and 61.9% for ART. However, the *p* values of 1.000 show that there is no statistically significant difference between the treatments (ART vs. SMART), suggesting that the treatment does not significantly influence success or failure in Class I and Class II cavities (Table 4).

**Table 4:** Success and failure rates in ART and SMART restorations among Class I and Class II cavities at 6 months.

	Treatment	Outcome at 6 months				*p*
		Success		Failure		
		*n*	%	*n*	%	
Class I (*n* = 23)	ART (*n* = 13)	10	76.90	3	23.10	1
	SMART (*n* = 10)	8	80	2	20	
Class II (*n* = 45)	ART (*n* = 21)	13	61.90	8	38.10	1
	SMART (*n* = 24)	16	66.60	8	33.40	

*Note:* Fisher's exact test.

### 3.4 Changes in OHRQoL After Both Treatments

A paired *t*‐test revealed statistically significant improvement in two items of the Child Impact Section (CIS): difficulty in chewing food and eating hot and cold beverages. Additionally, two items of the Family Impact Section FIS, “felt guilty” and “felt upset,” showed significant improvements (Table 5).

**Table 5:** Descriptive statistics for responses to ECOHIS questionnaire.

	Time of measurement				*p*
	At baseline		After 6 months		
	Mean	Standard deviation	Mean	Standard deviation	
**Child impact section (CIS)**					
(1) Pain in the teeth, mouth, or jaws	1.72	1	1.47	10	0.089
(2) Difficulty drinking hot or cold beverages	1.26	1	0.85	1	0.000*
(3) Difficulty to chew food	1.44	1	0.66	1	0.000*
(4) Difficulty pronouncing words	0.59	1	0.52	1	0.074
(5) Missed preschool or school	0.69	1	0.58	1	0.067
(6) Trouble sleeping	0.56	1	0.48	0.25	0.125
(7) Irritable or frustrated	0.98	1	0.88	1	0.329
(8) Avoid smiling or laughing	1	2	0.95	1	0.786
(9) Avoid talking when around other children	0.92	1	0.78	1	0.096
**Family impact section (FIS)**					
(10) Felt upset	1.65	1	0.92	1	0.000*
(11) Felt guilty	1.39	1	0.75	1	0.000*
(12) Time off work	0.72	1	0.53	1	0.365
(13) Financial impact	0.94	1	0.76	1	0.252

*Note:* Paired *t*‐test.

* Significant at *p* ≤ 0.05.

Overall, the mean and standard deviation for CIS were 9.16 ± 6.097 at baseline and decreased to 7.17 ± 5.051 at 6 months. For FIS, the mean and standard deviation values were 4.7 ± 5.308 at baseline and decreased to 2.96 ± 3.308 at 6 months. The mean and standard deviation values for the total ECOHIS score were 13.86 ± 6.98 at 3 months, dropping to 10.13 ± 6.13 at 6 months. However, no significant changes in the total CIS (*p* value = 0.085), FIS (*p* value = 0.074), and total ECOHIS (*p* value = 0.125) were observed (Table 6).

**Table 6:** Descriptive statistics for CIS, FIS, and total ECOHIS.

	Minimum	Maximum	Mean	Std. Deviation	*p*
CIS at baseline	3	17	9.16	6.097	0.085
CIS at 6 months	1	14	7.17	5.051	
FIS at baseline	2	9	4.7	3.308	0.074
FIS at 6 months	1	7	2.96	3.481	
Total ECOHIS at baseline	5	26	13.86	6.98	0.125
Total ECOHIS at 6 months	3	21	10.13	6.13	

*Note:* Paired *t*‐test.

## 4 Discussion

Children often experience anxiety during dental procedures due to fear of anesthesia, long treatment duration, and the use of multiple instruments, which can make treatment delivery challenging (Corrêa‐Faria et al. 2020). Therefore, MID provides a less invasive, less technique‐sensitive alternative that can help reduce anxiety and improve treatment tolerance in young patients (Wakhloo et al. 2021).

The current study compared the clinical performance of two minimal intervention techniques: SMART and ART in occlusal and occluso‐proximal cavities in primary molars after 6 months of follow‐up. Since both SMART and ART restorations exhibited comparable success rates, the first null hypothesis of this study is retained, as there is no significant difference in the clinical outcomes of ART and SMART, except for the superior caries‐arresting effect of SDF.

This split‐mouth study design was chosen to reduce variability, enhance accuracy, and minimize participant numbers by using each patient as their own control, while also addressing ethical considerations (Pandis et al. 2013). The study used 38% SDF for its proven effectiveness in arresting caries in primary teeth (Fung et al. 2016), and highly viscous glass ionomer cement (HVGIC) as the restorative material due to its suitability for primary molars (De Amorim et al. 2018). To avoid SDF‐induced staining, mixed HVGIC (non‐light cured) was used (Natarajan 2022). Restorations were evaluated using the Modified ART Criteria, which emphasize marginal integrity, wear, and secondary caries, making them suitable for preventive dentistry—unlike other restoration‐focused indices (Farag et al. 2011).

Children with Frankl scores of 2 and 3 were included, as they demonstrate sufficient tolerance for MID treatments, whereas definitely uncooperative children were directed to alternative treatment modalities, and those who were definitely cooperative received conventional restorative treatment.

At 6 months, no statistically significant difference in the overall success outcomes between the two treatments. This aligns with the results found by Aly et al. (2023), Boonyawong et al. (2020), and Jiang et al. (2020).

Moreover, the similar rates of wear and marginal failures for both restorations suggest that prior SDF treatment did not notably influence the success or mechanical performance of GIC. This finding aligns with Jiang et al. (2020), who found no significant impact of SDF on the performance of ART restorations. Similarly, De Amorim et al. (2018) reported that although ART and SMART offer certain benefits, they do not completely eliminate the risk of wear or marginal integrity challenges in molars.

In the present study, Class I restorations exhibited higher success rates than Class II restorations in both ART and SMART groups at the 6‐month follow‐up. This finding aligns with previous studies reporting that restoration success of GIC restorations is influenced by cavity type, with single‐surface restorations generally demonstrating superior clinical performance and longevity compared to multi‐surface restorations (Jiang et al. 2020; De Amorim et al. 2018; Giacaman et al. 2018).

Although failure rates were similar, Among the failed cases, active caries was detected only in ART restorations, whereas SMART restorations showed no signs of caries activity, in agreement with Satyarup et al. (2022) and Jiang et al. (2020), who reported that including the use of SDF, might provide additional protection against caries, which could explain the lower incidence of active caries in SMART restorations. Therefore, the presence of SDF under ART restoration in the SMART technique enhances the antibacterial properties and remineralization of GIC and helps to improve ART's ability to prevent caries progression (Jiang et al.^2020^).

It's noteworthy that, even in cases where GIC restorations in SMART technique were lost, caries remained arrested, consistent with the findings of Satyarup et al. (2022) and Jiang et al. (2020). This outcome is likely attributed to the remineralizing effect of 38% SDF, which effectively arrests caries progression. This action is associated with its alkaline pH, high fluoride concentration, and silver content, which together promote the formation of insoluble protective layers such as silver chloride, calcium fluoride, and metallic silver on demineralized enamel and dentin (Mei et al. 2018). Additionally, Seto et al. (2020) reported that silver microwires formed within the carious dentin fill structural voids, diffuse through dentinal tubules, and reinforce the lesion by distributing forces into sound dentin (Seto et al. 2020). This mechanism may explain the presence of hard, arrested surfaces without soft carious tissue, even when SMART restorations were partially or completely lost.

Furthermore, cases of pulp pathology emerged among failed ART restorations, likely due to the loss of the GIC filling material, which left open cavities where caries progression led to pulp infection.

In our trial, the same‐day SMART technique was implemented to reduce the number of visits for children. Unlike the multiple‐day appointments used in other studies. For instance, Mohammad et al.'s study, ART restorations were performed 1 week after SDF application, while in Jiang et al.'s study, they were placed 10 weeks later. Despite these differences, our findings remained consistent with theirs, showing no significant variation in outcomes (Mohammed et al.^2022^). This indicates that the timing of SDF application prior to dentin caries treatment may not affect the success of SMART restorations, although further research is needed.

The ECOHIS is the most commonly used tool for assessing OHRQoL in children under five, known for its reliability, responsiveness, and wide cultural adaptation into 14 languages, including Arabic (A‐ECOHIS) (Farsi et al. 2018). In this study, the effects of ART and SMART on OHRQoL were evaluated, with an initial mean ECOHIS score of 13.8 ± 6.98, indicating a negative impact of ECC on children's quality of life.

Improvements in certain items of CIS and FIS suggest that GIC restorations reduced children's discomfort while eating and drinking, likely by sealing dentin and preventing food impaction. Additionally, parents reported feeling less guilt and distress, possibly due to the relief of addressing their child's dental issues despite financial challenges. However, our study found that the total CIS, FIS, and overall ECOHIS were not affected after the placement of both restorations, and that aligns with the studies by Jiang et al. (2019) and Leal et al. (2013).

These limited changes in children's OHRQoL may be due to the minimally invasive nature of ART and SMART treatments, and a short follow‐up period may not be enough, which parents may not have noticed.

Studies indicate that ECOHIS is more sensitive to complex dental treatments like extractions and endodontic procedures than to nonoperative care (Novaes et al. 2017), with significant score reductions observed after treatment under general anesthesia (Faheem et al. 2023; Park et al. 2018).

The study has some limitations, such as the small sample size of 68 molars and a short follow‐up period of only 6 months, and this also applies to the ECOHIS questionnaire. Additionally, patient‐related factors, such as oral hygiene and dietary habits, which could influence the success of the restorations, were not evaluated. Moreover, blinding the operator to the treatment groups was not feasible, as SDF stains the dentin under GIC fillings, making it easily identifiable. Future studies with larger samples and longer follow‐up periods are recommended for more significant results.

## 5 Conclusion

Within the limitations of our study, both ART and SMART restorations show similar clinical outcomes; however, SDF is particularly effective in arresting caries and plays a crucial role in improving the effectiveness of the ART technique in caries management. Additionally, the higher failure rate of ART restorations due to active caries and pulp issues suggests that adding SDF can help maintain caries arrest after GIC's fluoride effect fades.

## Author Contributions

**Sana K. Solh:** conceptualization, methodology, data curation, writing – original draft. **Ahmad S. Tarabaih:** supervision, methodology, resources, investigation, writing – review and editing. **Ahmed A. Holiel:** supervision, methodology, data curation, validation, writing – review and editing.

## Funding

The authors received no specific funding for this work.

## Ethics Statement

This study was approved by the Institutional Review Board of Beirut Arab University (IRB code: 2022‐H‐011‐D‐M‐0489).

## Consent

Written informed consent was obtained from the parents or legal guardians of all participating children in accordance with institutional guidelines.

## Conflicts of Interest

The authors declare no conflicts of interest.

## Data Availability

The data that support the findings of this study are available on request from the corresponding author. The data are not publicly available due to privacy or ethical restrictions.
